# Intracellular expression of Tat alters mitochondrial functions in T cells: a potential mechanism to understand mitochondrial damage during HIV-1 replication

**DOI:** 10.1186/s12977-015-0203-3

**Published:** 2015-09-16

**Authors:** Sara Rodríguez-Mora, Elena Mateos, María Moran, Miguel Ángel Martín, Juan Antonio López, Enrique Calvo, María Carmen Terrón, Daniel Luque, Delphine Muriaux, José Alcamí, Mayte Coiras, María Rosa López-Huertas

**Affiliations:** Unidad de Inmunopatología del SIDA, Centro Nacional de Microbiología, Instituto de Salud Carlos III, Majadahonda, Madrid, Spain; Laboratorio de Enfermedades Raras: mitocondriales y neuromusculares, Instituto de Investigación Hospital 12 de Octubre, “i + 12”, Madrid, Spain; Centro de Investigación Biomédica en Red de Enfermedades Raras (CIBERER) U723, Madrid, Spain; Unidad de Proteómica, Centro Nacional de Investigaciones Cardiovasculares, Madrid, Spain; Unidad de Microscopía Electrónica y Confocal, Centro Nacional de Microbiología, Instituto de Salud Carlos III, Majadahonda, Madrid, Spain; Unité de Virologie Humaine - INSERM U758/École Normale Supérieure, Lyon, France; Laboratoire de Domaines Membranaires et Assemblage Viral, Centre d’études d’agents Pathogènes et Biotechnologies pour la Santé, Montpellier, France

**Keywords:** HIV-1, Tat, Mitochondria, Cytoskeletal rearrangements, Aerobic glycolysis, mtDNA transcription

## Abstract

**Background:**

HIV-1 replication results in mitochondrial damage that is enhanced during antiretroviral therapy (ART). The onset of HIV-1 replication is regulated by viral protein Tat, a 101-residue protein codified by two exons that elongates viral transcripts. Although the first exon of Tat (aa 1–72) forms itself an active protein, the presence of the second exon (aa 73–101) results in a more competent transcriptional protein with additional functions.

**Results:**

Mitochondrial overall functions were analyzed in Jurkat cells stably expressing full-length Tat (Tat101) or one-exon Tat (Tat72). Representative results were confirmed in PBLs transiently expressing Tat101 and in HIV-infected Jurkat cells. The intracellular expression of Tat101 induced the deregulation of metabolism and cytoskeletal proteins which remodeled the function and distribution of mitochondria. Tat101 reduced the transcription of the mtDNA, resulting in low ATP production. The total amount of mitochondria increased likely to counteract their functional impairment. These effects were enhanced when Tat second exon was expressed.

**Conclusions:**

Intracellular Tat altered mtDNA transcription, mitochondrial content and distribution in CD4+ T cells. The importance of Tat second exon in non-transcriptional functions was confirmed. Tat101 may be responsible for mitochondrial dysfunctions found in HIV-1 infected patients.

**Electronic supplementary material:**

The online version of this article (doi:10.1186/s12977-015-0203-3) contains supplementary material, which is available to authorized users.

## Background

Human immunodeficiency virus type 1 (HIV-1) infection is characterized by a progressive depletion of CD_4_^+^ T lymphocytes in the peripheral blood and lymphoid organs which finally leads to the onset of acquired immune deficiency syndrome [1]. HIV-1 replication results in the chronic activation of the immune system and consequently in premature immunosenescence [2]. During normal aging, mitochondrial DNA (mtDNA) accumulate mutations induced through the direct exposition to reactive oxygen species (ROS) and deficient repair systems [3, 4]. Accordingly, T CD4+ lymphocytes from naïve HIV-1 infected patients show mtDNA depletion along with impaired activity of the respiratory chain components, enhanced oxidative damage and decrease mitochondrial transmembrane potential (∆ψm) [5, 6]. These alterations may be explained by a deregulated expression of nuclear and mitochondrial encoded proteins such as Prohibitin, Heat shock protein 60 (Hsp60) and complex-I subunit NDUF6 [7, 8]. Furthermore, it is well-known that the antiretroviral treatment (ART) against HIV-1, in particular nucleoside-analogue inhibitors (NRTIs), induces mitochondrial toxicity [9, 10] due to the inhibition of mtDNA polymerase γ [11]. In summary, HIV-1 infection triggers mitochondria impairment aside from the toxicity of the treatment and therefore it may enhance the susceptibility for the clinical onset of ART side-effects.

HIV-1 Tat is a 101-residue protein codified by two exons that promotes the efficient elongation of the viral transcripts through the binding to RNA polymerase II (RNAPII) complex and the recruitment of cellular elongation factors [12]. The first exon of Tat includes amino acids (aa) 1–72 and encodes an active protein that partially retains the elongation ability of full-length Tat [13]. The second exon is codified by amino acids 73–101 and its expression within the protein results in a more competent transcriptional protein with additional functions independent of transcription such as control of apoptosis [14, 15]. Tat can be released from infected cells and up-taken by non-infected adjacent cells. Extracellular and intracellular Tat frequently have opposite actions, as Tat expressed inside the host-infected cells can delay apoptosis upon FasL-challenge [14–17], but it is a potent death inductor in its soluble form [18, 19]. Although loss of ∆ψm and increased levels of ROS has been found after Tat extracellular exposure [20–22], few data are available about the effect of intracellular Tat on mitochondria functions. Our group showed that intracellular Tat deregulated the expression of proteins required for mitochondrial membrane stabilization as HSP and Prohibitin [15].

In this work, we describe the single influence of intracellular HIV-1 Tat protein on mitochondria overall functions in CD4+ T cells, showing that Tat101 remodeled mitochondria distribution and enhanced the mitochondria content probably to compensate the mtDNA transcription impairment. These effects were enhanced when Tat second exon was expressed.

## Results

### Tat101 deregulated the expression of cellular proteins related to metabolism and oxidative stress

**Fig. 1:**
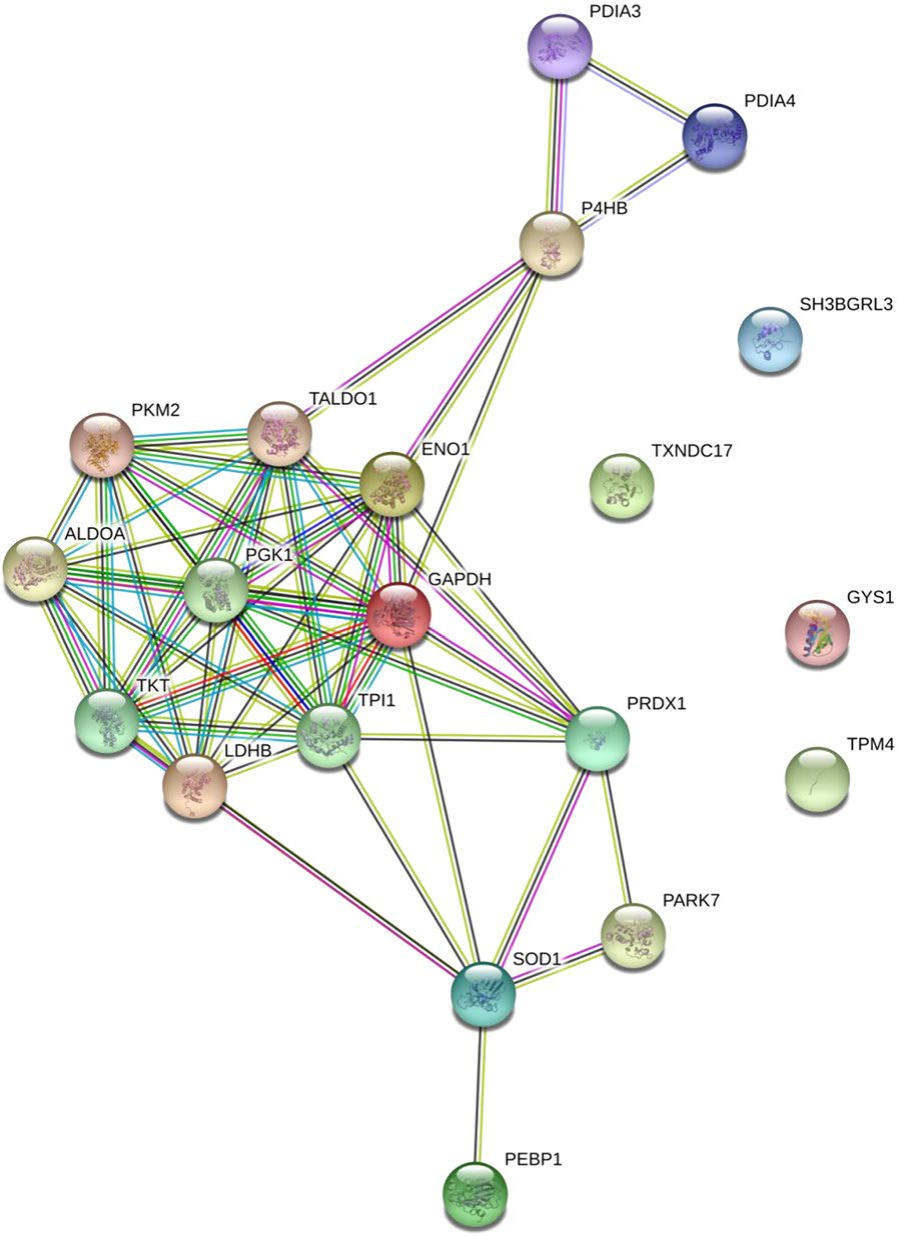
Network of predicted interactions between mitochondria-related proteins deregulated in Jurkat-Tat101. Medium confidence score level was 0.400. Data supporting protein–protein interactions derived from experimental studies (*dark purple lines*), homology (*light purple lines*), databases (*light blue lines*), text mining (*light green lines*), concurrence (*dark blue lines*) and co-expression (*black lines*). Node *colour* is arbitrary. Differences in protein levels are specified in Table 1

To describe the role of Tat on mitochondria, Jurkat cells stably expressing Tat72 (Jurkat-Tat72) or Tat101 (Jurkat-Tat101) protein were used as a proper model of CD4+ T lymphocytes as previously shown [15]. The proteome of Jurkat-Tat101 and Jurkat-Tat72 was analyzed by liquid chromatography–mass spectrometry (LC–MS)/MS in basal conditions, in comparison with control cells. A comparative analysis of the LC–MS/MS proteome showed that the intracellular expression of Tat101 deregulated the expression of 19 cellular proteins involved in metabolism and control of oxidative stress (Table 1). A total of 14 proteins were up-regulated, representing 73.68 % of the deregulated proteins. To a lower extend, these proteins were also deregulated in Jurkat-Tat72 cells. Proteins with major functions in glycolysis included alpha-enolase (ENO1), glyceraldehyde-3-phosphate dehydrogenase (GAPDH), l-lactate dehydrogenase (LDHB) and pyruvate kinase isozymes M1/M2 (PKM2), which were, respectively 17.9-, −11.4-, 10.4- and 36.8-fold expressed in Jurkat-Tat101 cells versus control cells. It was found an overall up-regulation of proteins from the thioredoxin and glutaredoxin redox superfamilies. These proteins included protein disulfide-isomerase (P4HB), thioredoxin domain-containing protein 17 (TXDC17), protein disulfide-isomerase A4 (PDIA4) and SH3 domain-binding glutamic acid-rich-like protein 3 (SH3BGR3), which were, respectively, 2.5-, 2.8-, 5.2-, and 8.4-fold deregulated. Other proteins related to the oxidative metabolism such as superoxide dismutase (SOD1) and peroxiredoxin-1 (PRDX1) were 3.3- and 7.8-fold up-regulated in Jurkat-Tat101 cells. Most of these proteins were functionally interconnected as it was observed after analyzing the predicted interactions by STRING database (Fig. 1).

**Table 1:** Selection of mitochondrial-related proteins which showed at least a ±2.0-fold deregulation in Jurkat-Tat101 versus control cells

Gene	Protein	Tat72 vs TetOff	Tat101 vs TetOff	Biological process	Peptide sequence	X-corr
PKM2	Pyruvate kinase isozymes M1/M2	6.5	36.8	Glycolysis	APIIAVTRNPQTAR EAEAAIYHLQLFEELRR FGVEQDVDMVFASFIR GSGTAEVELKK IYVDDGLISLQVK KGVNLPGAAVDLPAVSEKDIQDLK LDIDSPPITAR RFDEILEASDGIMVAR SVETLKEMIK TATESFASDPILYRPVAVALDTK TATESFASDPILYRPVAVALDTKGPEIR	2.65 1.81 2.90 2.06 1.88 3.72 2.01 3.16 1.96 2.80 3.18
LDHB	l-lactate dehydrogenase (B chain)	−0.9	10.4	Glycolysis	IVADKDYSVTANSK LKDDEVAQLKK SADTLWDIQK SADTLWDIQKDLKDL	2.07 2.26 2.05 2.08
SH3BGRL3	SH3 domain-binding glutamic acid-rich-like protein 3	9.9	8.1	Oxidative stress	IQYQLVDISQDNALRDEMR VYSTSVTGSR	3.12 2.48
PRDX1	Peroxiredoxin-1	7.9	7.8	Oxidative stress	QGGLGPMNIPLVSDPKR QITVNDLPVGR TIAQDYGVLKADEGISFR	1.78 2.06 2.95
PDIA4	Protein disulfide-isomerase A4	0.7	5.2	Oxidative stress	FDVSGYPTIK FDVSGYPTLK IDATSASVLASR MDATANDVPSDR VDATAETDLAKR	1.98 1.98 2.51 1.75 1.83
TKT	Transketolase	−1.2	4.9	Energy metabolism	ILATPPQEDAPSVDIANIR KAYGQALAK MFGIDRDAIAQAVR	1.80 2.29 2.71
TXNDC17	Thioredoxin domain-containing protein 17	0.9	2.8	Oxidative stress	YEEVSVSGFEEFHR	1.77
SOD1	Superoxide dismutase	0.4	3.3	Oxidative stress	TLVVHEKADDLGK TLVVHEKADDLGKGGNEESTK	1.76 3.07
ALDOA	Fructose-bisphoshate aldolase A	8.9	2.7	Glycolysis	AAQEEYVKR GILAADESTGSIAK GILAADESTGSIAKR IGEHTPSALAIMENANVLAR IVAPGKGILAADESTGSIAK IVAPGKGILAADESTGSIAKR LQSIGTENTEENR	1.88 3.03 2.61 2.69 3.80 3.91 2.41
P4HB	Protein disulfide-isomerase	1.5	2.5	Oxidative stress	MDSTANEVEAVKVHSFPTLK	2.02
GYS1	Glycogen synthase, muscle	0.9	2.5	Glycogenesis	LSDLLDWK TQVELLEAPTPALKR VGGIYTVLQTK	1.79 1.94 1.87
TPM4	Tropomyosin alpha-4	9.9	2.2	Oxidative stress	KIQALQQQADEAEDR	2.13
TALDO1	Transaldolase	0.6	2.2	Energy metabolism	WLHNEDQMAVEK	2.15
PEBP1	Phosphatidylethanolamine-binding protein	0.0	2.2	Oxidative stress	LYEQLSGK WSGPLSLQEVDEQPQHPLHVTYAGAA	1.79 2.68
PARK7	Protein DJ-1	1.8	−2.0	Autophagy Fusion Morphogenesis	GAEEMETVIPVDVMR GAEEMETVIPVDVMRR	2.08 2.00
PGK1	Phosphoglycerate kinase 1	1.3	−2.7	Glycolysis	AHSSMVGVNLPQK LGDVYVNDAFGTAHR VDFNVPMKNNQITNNQR VLPGVDALSNI	1.81 2.60 2.00 1.96
TPI1	Triosephosphate isomerase isoform 1	−5.3	−3.0	Glycolysis gluconeogenesis	SNVSDAVAQSTR VVLAYEPVWAIGTGK	2.95 2.65
GAPDH	Glyceraldehyde-3-phosphate dehydrogenase	−10.8	−11.4	Glycolysis	GALQNIIPASTGAAK IKWGDAGAEYVVESTGVFTTMEK LISWYDNEFGYSNR VGVNGFGR VVDLMAHMASKE WGDAGAEYVVESTGVFTTMEK	1.81 2.17 3.07 2.09 2.87 2.53
PDIA3	Protein disulfide-isomerase A3	−15.0	−11.9	Oxidative stress	DGEEAGAYDGPR EATNPPVIQEEKPK ELSDFISYLQR GFPTIYFSPANK IFRDGEEAGAYDGPR KYEGGRELSDFISYLQR LSKDPNIVIAK MDATANDVPSPYEVR QAGPASVPLR QAGPASVPLRTEEEFKK RLAPEYEAAATR TADGIVSHLKK YGVSGYPTLK	2.09 2.30 2.43 2.34 3.09 2.91 2.14 2.62 2.59 1.82 2.68 2.68 2.05

Proteins were detected by mass spectrometry in total protein extracts. All peptides detected showed 95 % probability for protein expression and a minimum cross-correlation (X-Corr) value of 1.75

### Tat101 increased citrate synthase activity and lactate levels but reduced the activity of respiratory complexes

**Fig. 2:**
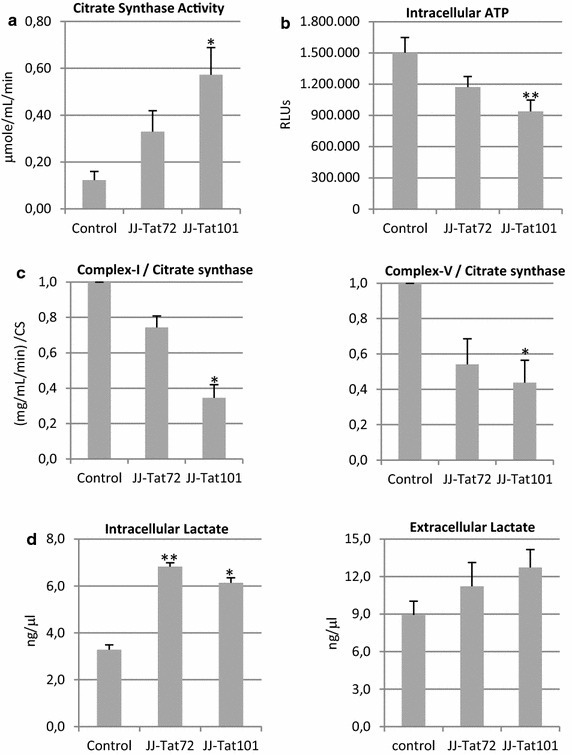
Quantification of citrate synthase activity, mitochondria respiratory capacity and lactate levels in Jurkat-Tat101 cells. **a** Activity of the citrate synthase measured with commercial enzymatic assays in Jurkat-Tat72 (JJ-Tat72), Jurkat-Tat101 (JJ-Tat101) vs control cells. One citrate unit is equivalent to a μmole/mL/min. **b** ATP production was measured using a chemiluminescence-based assay. **c** Activity of the complex-I and complex-V of the respiratory chain was measured in Jurkat-Tat72, Jurkat-Tat101 and control cells in mg/mL/min and it was normalized according to mitochondria amount indirectly measured as the activity of the citrate synthase enzyme. **d** Lactate levels were measured in intracellular and supernatant samples and concentration was expressed in ng/μl (*left* and *right panels*, respectively) from Jurkat-Tat72, Jurkat-Tat101 vs control cells. All data shown are media and standard error of the mean (SEM) from at least three independent experiments. Statistical significance was calculated by Kruskal–Wallis test and post hoc multiple comparisons were performed with Dunn’s multiple comparison analysis (*p < 0.05 and **p < 0.01 vs control)

Citrate synthase levels are routinely used as a marker mitochondrial content [23]. In Jurkat-Tat101 cells, citrate synthase activity increased 4.75-fold regarding control cells (*p* < 0.05), whereas it increased only 2.75-fold in Jurkat-Tat72 (Fig. 2a). Although the average amount of mitochondria was increased, the overall production of ATP was significantly reduced in Jurkat-Tat101 cells (*p* < 0.01) (Fig. 2b), suggesting that these mitochondria were not functionally competent. To test this hypothesis, the enzyme activity of complex-I and complex-V of the respiratory chain was measured, using citrate synthase activity to normalize mitochondrial content. Complex-I and complex-V activities were 2.85- and 2.27-fold reduced, respectively, in Jurkat-Tat101 (*p* < 0.05). Complex-I and complex-V were only 1.35- and 1.85-fold reduced in Jurkat-Tat72, respectively (Fig. 2c).

As the function of complex-I and complex-V complexes was impaired in Jurkat-Tat101 cells and ATP production was reduced but not completely stopped, aerobic glycolysis was studied as a complementary energy source in Jurkat-Tat101 cells. Aerobic glycolysis is a metabolic program in which the lactate production occurs in the presence of oxygen [24, 25]. Intracellular levels of lactate were measured under aerobic conditions and both Jurkat-Tat101 and Jurkat-Tat72 cells showed a similar increase of 2.0-fold (*p* < 0.05) (Fig. 2d, left graph). A slight non-significant increase of 1.25- and 1.42-fold of lactate levels was found in the culture medium of Jurkat-Tat72 and Jurkat-Tat101 cells, respectively, in comparison to control cells (Fig. 2d, right graph).

### Enhanced ROS production was counteracted by glutathione system in Jurkat-Tat cells

**Fig. 3:**
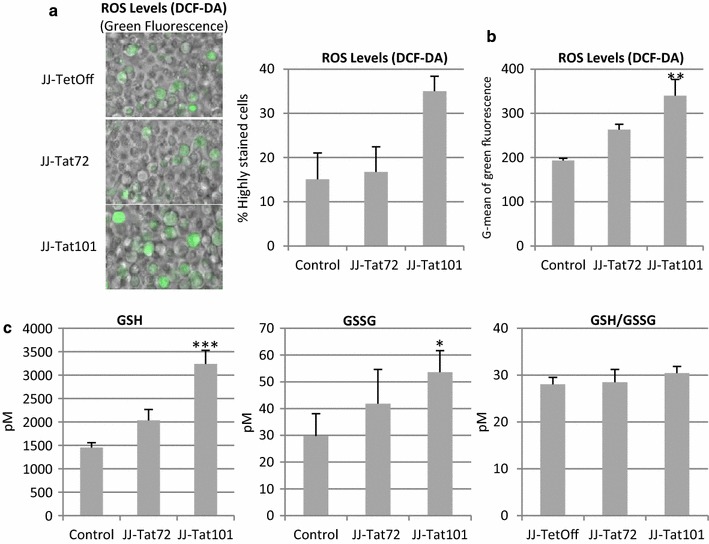
Intracellular ROS generation and glutathione levels in Jurkat-Tat101 cells. **a** Microscopy analysis of intracellular ROS levels measured by DCF-DA staining method. Representative fields of living Jurkat-Tat72, Jurkat-Tat101 and control cells are shown. Acquisition conditions remained the same for each cellular type. The *graph* shows the number of cells with saturated signal for green laser, from three independent experiments. **b** Cytometry analysis of DCF-DA stained cells. *Graph* shows G-mean of green fluorescence intensity of the living cell population from three independent experiments. **c** Intracellular concentration of reduced (GSH), oxidized (GSSG) and ratio of total glutathione (GSH/GSSG) were measured in Jurkat-Tat72, Jurkat-Tat101 and control cells. Data shown are media and SEM from at least three independent experiments. Kruskal–Wallis test with Dunn’s Multiple Comparison post hoc analysis was performed for statistical analysis (*p < 0.05 and ***p < 0.001 vs control)

A enhanced production of ROS in productively and latently HIV-1 infected CD4+ T cells has been described [5]. Intracellular levels of ROS were measured using dichlorofluorescein diacetate (DCF-DA) staining method [26]. Immunofluorescence analysis showed that 35.0 % of Jurkat-Tat101 cells showed high DCF-DA signal, whereas both Jurkat-Tat72 and control cells showed only 15 % of highly stained cells (Fig. 3a). Acquisition conditions remained and a representative field of living Jurkat-Tat72 and Jurkat-Tat101 is shown. Similarly, flow cytometry quantification of the geometry mean (G-mean) of green fluorescence increased 1.8-fold in Jurkat-Tat101 and 1.4-fold in Jurkat-Tat72 cells, regarding control cells (*p* < 0.01) (Fig. 3b).

Glutathione is an abundant antioxidant that mostly exits in its reduced form (GSH). A small percentage of the glutathione is oxidized (GSSG) and acts as an indicator of oxidative stress. The intracellular levels of GHS and GSSG were enhanced, respectively 2.2-fold and a 1.8-fold in Jurkat-Tat101 (*p* < 0.005 and *p* < 0.05) (Fig. 3c). In Jurkat-Tat72 cells, the increase of glutathione levels was lower than in Jurkat-Tat101 cells and did not reach statistical significance. The GSH/GSSG ratio that measures the cellular oxidative stress [27] remained unaltered in Jurkat cells expressing any isoform of Tat protein.

### Intracellular expression of Tat101 increased basal apoptosis

**Fig. 4:**
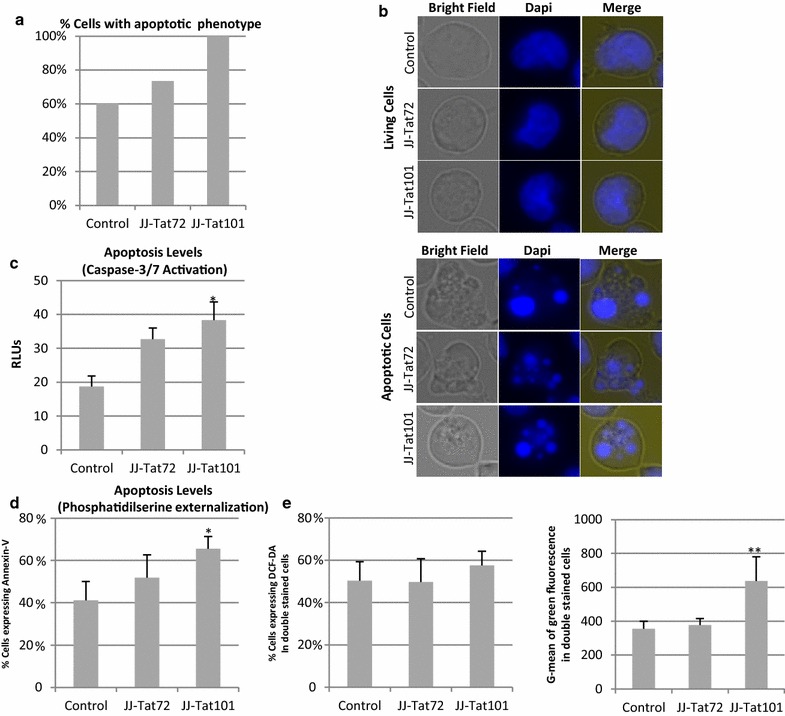
Apoptosis induction in non-stimulated Jurkat-Tat101 cells. **a** Percentage of Jurkat-Tat72, Jurkat-Tat101 and control cells showing an apoptotic phenotype from three independent experiments analyzed by confocal microscopy. **b** Representative images of living and apoptotic cells. Cells were fixed and the nuclei were stained with Dapi. **c** Caspase-3/-7 activation was measured by a chemiluminiscence-based test in Jurkat-Tat72, Jurkat-Tat101 and control cells under basal conditions. Relative luciferase units (RLUs) after total protein normalization are shown. **d** Percentage of apoptosis committed cells expressing external phosphatidylserine. The *graph* shows the cytometry analysis of Annexin-V-PE stained cells. **e** Cytometry analysis of doubly stained cells for Annexin-V-PE and DCF-DA-FITC. *Graph* on the *left* shows the percentage of double stained cells. *Graph* on the *right* shows G-mean of green fluorescence intensity in double stained cells. All *graphs* show media and SEM from at least three independent experiments. Kruskal–Wallis test with Dunn’s multiple comparison post hoc analysis was performed for statistical analysis (*p < 0.05, **p < 0.001)

The intracellular expression of Tat101 protein delays Fas-mediated apoptosis during HIV-1 infection in CD4+ T lymphocytes [15]. In this work, we show that the percentage of cells displaying a typical apoptotic phenotype, i.e. morphological disintegration and bubbling of the plasma membrane, increased 2.0-fold in Jurkat-Tat101 cells and to a lesser extent also in Jurkat-Tat72 cells, vs control cells (Fig. 4a). It was observed in basal conditions and in the absence of activation or death stimuli. Selected cells with representative living and apoptotic phenotypes are shown in Fig. 4b. Activation of the effector caspase-3/-7 under basal conditions correlated with apoptotic phenotypes and it was 1.8- and 2.0-fold higher in Jurkat-Tat72 and Jurkat-Tat101 cells, respectively, than in control cells (Fig. 4c) (*p* < 0.05).

Apoptosis commitment was measured by quantifying externalization of phosphatidylserine (PS) in Annexin-V stained cells and subsequent flow cytometry analysis, showing a 1.4-fold increase in Jurkat-Tat101 versus control cells (*p* < 0.05) (Fig. 4d). As increased ROS production occurred occurs during cell death, co-localization of intracellular ROS and PS externalization was studied in control and Jurkat-Tat expressing cells doubled stained with DCF-DA and Annexin-V (Fig. 4e). Around 50 % of apoptosis-committed cells expressed intracellular ROS (Fig. 4e, left panel). However, G-mean of green fluorescence increased 1.8-fold in Jurkat-Tat101 cells doubly stained for ROS and apoptosis, regarding control cells (*p* < 0.01) (Fig. 4e, right panel). These data support the enhancement of intracellular ROS and apoptosis independently showed in Figs. 3 and 4 (panels from a to d), respectively.

### Tat101 reduced the transcription of mtDNA encoded genes

**Fig. 5:**
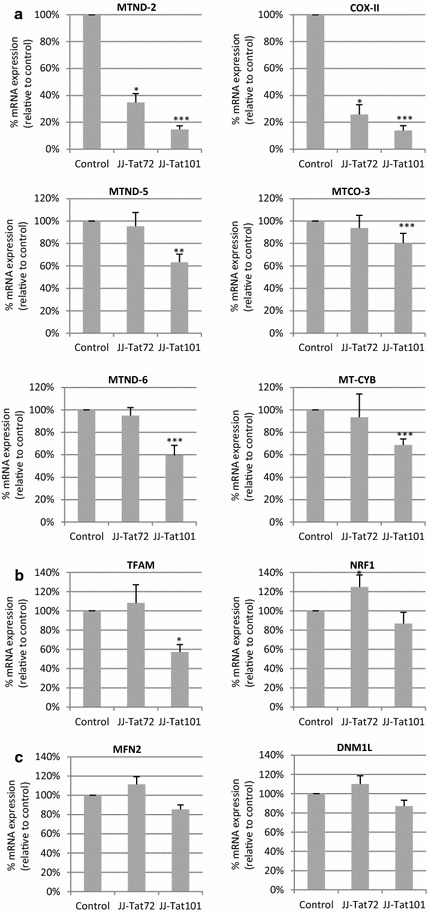
Effect of Tat on the transcription of mt-DNA and fusion/fission genes. **a** qPCR analysis of mRNAs levels from the mtDNA-encoded genes COX-II, MTND-2, MTND-5, MTND-6, MT-CYB and MTCO-3. **b** qRT-PCR analysis of mRNAs levels from nuclear DNA-encoded genes TFAM and NRF1. **c** qRT-PCR analysis of nuclear-encoded mRNAs from MFN2 and DNM1L genes, which are involved in mitochondria fusion and fission, respectively. Total mRNA from control cells, Jurkat-Tat72, and Jurkat-Tat101 cells was analyzed. Nuclear S18 mRNA expression was used as house-keeping gene. All data shown are media and SEM from at least three independent experiments. Statistical significance was calculated by Kruskal–Wallis test with Dunn’s multiple comparison post hoc analysis (*p < 0.05, **p < 0.01 ***p < 0.001 vs control)

A unique feature of mitochondria is that they contain their own 16-kb genome that encodes mitochondria components including 13 subunits of the mitochondria respiratory chain essential for the mitochondrial oxidative phosphorylation (OXPHOS) [28, 29]. The transcription of six mtDNA encoded genes was studied in Jurkat-Tat expressing cells (Fig. 5a). These genes included components of the respiratory chain complex-I, complex-III and complex-IV and were NADH-ubiquinone oxidoreductase chain 2 (MTND-2), NADH-ubiquinone oxidoreductase chain 5 (MTND-5), NADH-ubiquinone oxidoreductase chain 6 (MTND-6), cytochrome *c* oxidase subunit 2 (COX-II), cytochrome *c* oxidase subunit 3 (MTCO-3), and cytochrome *b* (MT-CYB). The intracellular expression of Tat101 protein resulted in a significant reduction of more than 6.0-fold in both COX-II and MTND-2 mRNA levels (*p* < 0.005) (Fig. 5a). In Jurkat-Tat72 cells, COX-II and MTND-2 mRNA levels were 3.0-fold reduced (*p* < 0.05). Although to a lower extend, the expression of other mtDNA encoded genes were also significantly reduced in Jurkat-Tat101. MTND-5, MTND-6, MT-CYB andMTCO-3 mRNAs levels were around 1.5-fold reduced in Jurkat Tat-101 cells (*p* < 0.005 and *p* < 0.001) but not in Jurkat-Tat72 cells (Fig. 5a). These data show the down-regulation of mtDNA transcription.

The control of gene expression from mtDNA is directed by nuclear-encoded genes as respiratory factor 1 (*NRF*-*1*) and mitochondrial transcription factor A (*TFAM*) [30]. TFAM mRNA expression was 1.75-fold reduced in Jurkat-Tat101 cells (Fig. 5b) (*p* < 0.05). No significant differences were found in the expression of *NRF*-*1* mRNA in Jurkat-Tat101 cells but the levels of *NRF*-*1* mRNA were slightly enhanced in Jurkat-Tat72cells (Fig. 5b). mRNAs levels of nuclear-encoded genes involved in mitochondrial fusion and fission were analyzed and there were not significant changes in the expression of *MFN2* and *DNML1* mRNAs (Fig. 5c).

### Intracellular Tat101 enhanced the expression of nuclear-encoded mitochondrial genes

**Fig. 6:**
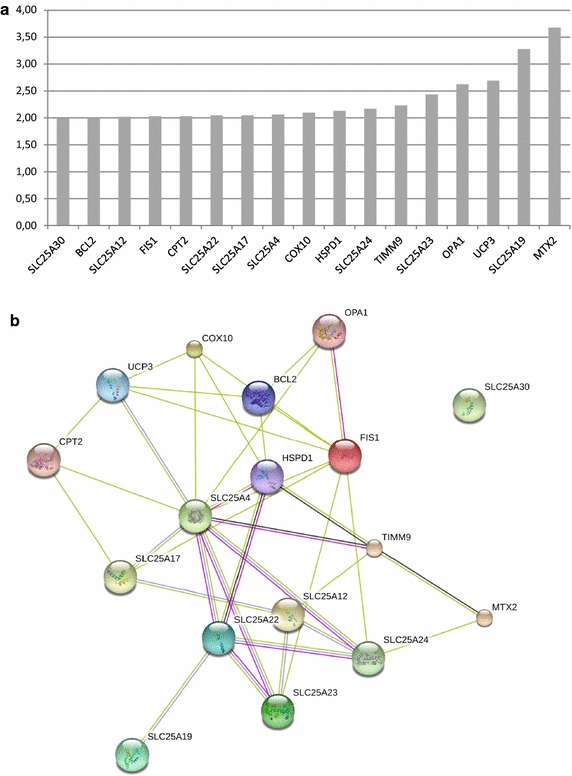
Expression of an array of nuclear-encoded genes related to mitochondria in Jurkat-Tat101 cells. The expression of mitochondrial genes encoded by nuclear DNA was analyzed by qRT-PCR in total RNA from Jurkat-Tat101 cells versus controls cells, using *RT* ^*2*^ *Profiler™ PCR Array Human Mitochondria.* **a** Relative expression levels of mitochondrial-related genes deregulated at least ±2.0-fold in Jurkat-Tat101 cells versus control cells. Table 2 includes the relative gene expression levels of 84 mitochondrial-genes included in the analysis. **b** Network of predicted interactions between mitochondria-related genes deregulated ±2.0-fold in Jurkat-Tat101. Medium confidence score level was 0.400. Data supporting protein–protein interactions derived from experimental studies (*dark purple lines*), homology (*light purple lines*), databases (*light blue lines*), text mining (*light green lines*), concurrence (*dark blue lines*) and co-expression (*black lines*). Node *colour* is arbitrary

Intracellular Tat101 protein is known to profoundly deregulate gene expression in T lymphocytes [14, 31]. Therefore the effect of Tat101 protein in the transcription of nuclear-encoded genes required for mitochondria functions was intensely described. A commercial RT-PCR-based array was used to compare the expression of 84 mitochondrial genes, which were nuclear-encoded, in Jurkat-Tat101 cells versus control cells. Table 2 shows C_T_ values and the relative expression of each gene in Jurkat-Tat101 versus control cells after normalizing to the housekeeping gene expressions and analyzing with the 2^−ΔΔCt^ formula. Genes showing C_T_ values equal or higher than 30 cycles or undetermined were not considered for additional analysis and therefore only the expression of 77 genes was studied. The expression levels of 67 genes were increased at least +1.5-fold in Jurkat-Tat101 cells, suggesting a generalized up-regulation of mitochondrial-related genes. These genes represent 88.5 % of the mitochondrial genes studied. A higher cutoff of ∓2.0-fold change yield 17 genes upregulated in Jurkat-Tat101 cells (Fig. 6a). There was an enhanced expression of eight members from mitochondrial solute carrier family 25 (SLC25), a superfamily of proteins that shuttle metabolites, nucleotides, and cofactors through the mitochondrial inner membrane including the exchange of cytoplasmic ADP with mitochondrial ATP [32]. For example, levels of SLC25A23 and SLC25A19 increased 2.43- and 3.28-fold, respectively. Other mitochondrial transporters deregulated included metaxin 2 (MTX2), involved in importing proteins into mitochondria [33], uncoupling protein 3 (UCP3), a proton transporter resulting in OXPHOS uncoupling also known as SLC25a9 [34], and translocase of inner mitochondrial membrane 9 (TIMM9), a chaperone involved in the import of transmembrane proteins [35], which were respectively 3.68-, 2.69- and 2.23-fold enhanced. The functional interconnection of these proteins was predicted in STRING database and is shown in Fig. 6b. These data suggest an increased expression of nuclear-encoded mitochondrial genes in Jurkat-Tat101 cells and confirm proteome results showed in Table 1 and Fig. 1.

**Table 2:** C_T_ and fold expression of mitochondrial nuclear-encoded genes in Jurkat-Tat101 cells

Gene	C_T_		Fold of expression	Gene	C_T_		Fold of expression
	JJ-control	JJ-Tat101	JJ-Tat101/JJ-control		JJ-control	JJ-Tat101	JJ-Tat101/JJ-control
AIFM2	26.97	29.28	0.85	SLC25A17	20.72	21.95	2.05
AIP	19.44	20.72	1.74	SLC25A19	21.54	22.58	3.28
BAK1	22.30	23.80	1.49	SLC25A2	28.58	28.95	1.90
BBC3	28.93	30.27	1.66	SLC25A20	22.77	23.92	1.68
BCL2	23.63	24.71	2.00	SLC25A21	27.02	28.34	1.87
BCL2L1	22.90	24.42	1.47	SLC25A22	23.78	24.95	2.05
BID	17.96	19.14	1.86	SLC25A23	25.51	26.55	2.43
BNIP3	18.59	19.68	1.98	SLC25A24	21.17	21.96	2.17
CDKN2A	Undetermined	Undetermined	1.83	SLC25A25	23.91	24.87	1.00
COX10	23.44	24.64	2.10	SLC25A27	37.15	37.16	4.19
COX18	23.24	24.24	1.21	SLC25A3	16.89	17.98	1.97
CPT1B	30.36	32.16	1.61	SLC25A30	23.34	24.41	2.00
CPT2	21.88	23.27	2.03	SLC25A31	32.62	32.78	3.77
DNM1L	20.42	21.47	1.58	SLC25A37	21.47	22.65	1.86
FIS1	19.89	21.31	2.03	SLC25A4	21.93	22.96	2.06
TIMM10B	21.92	22.98	1.73	SLC25A5	16.59	18.12	1.45
GRPEL1	19.53	20.81	1.80	SOD1	16.03	17.40	1.63
HSP90AA1	15.48	16.70	1.80	SOD2	19.48	20.69	1.82
HSPD1	16.17	17.39	2.13	STARD3	23.29	24.53	1.78
IMMP1L	20.91	21.90	1.52	TAZ	21.59	23.00	1.59
IMMP2L	22.01	23.48	1.99	TIMM10	17.79	19.19	1.59
LRPPRC	19.33	20.41	1.97	TIMM17A	19.38	20.67	1.71
MFN1	21.42	22.52	1.84	TIMM17B	20.85	22.26	1.59
MFN2	21.75	22.95	1.85	TIMM22	22.65	23.73	1.99
MIPEP	23.34	24.52	1.85	TIMM23	19.48	20.69	1.82
MPV17	21.24	22.43	1.61	TIMM44	21.38	22.68	1.70
MSTO1	22.30	23.68	1.59	TIMM50	19.19	20.29	1.96
MTX2	20.15	21.56	3.68	TIMM8A	20.40	21.65	1.77
NEFL	32.20	32.39	1.97	TIMM8B	17.59	18.94	1.65
OPA1	20.33	21.43	2.62	TIMM9	24.46	25.37	2.23
PMAIP1	21.54	22.22	1.73	TOMM20	17.88	19.14	1.75
RHOT1	20.37	21.66	1.72	TOMM22	17.92	19.41	1.50
RHOT2	21.68	22.97	1.73	TOMM34	20.85	22.18	1.67
SFN	25.74	27.02	1.95	TOMM40	20.69	22.37	1.31
SH3GLB1	20.52	21.63	1.47	TOMM40L	23.80	24.95	1.89
SLC25A1	19.77	21.29	1.04	TOMM70A	19.70	20.78	1.99
SLC25A10	25.56	27.57	1.84	TP53	22.63	23.91	1.73
SLC25A12	21.66	22.85	2.02	TSPO	30.82	34.61	0.31
SLC25A13	24.15	25.21	1.94	UCP1	26.98	28.95	1.07
SLC25A14	22.35	23.46	1.83	UCP2	19.92	21.44	1.46
SLC25A15	21.18	22.38	1.99	UCP3	27.19	27.84	2.69
SLC25A16	21.68	22.76	1.80	UXT	18.46	19.73	1.74

### Intracellular Tat101 deregulated the expression of cytoskeleton proteins and activated small GTPases

**Fig. 7:**
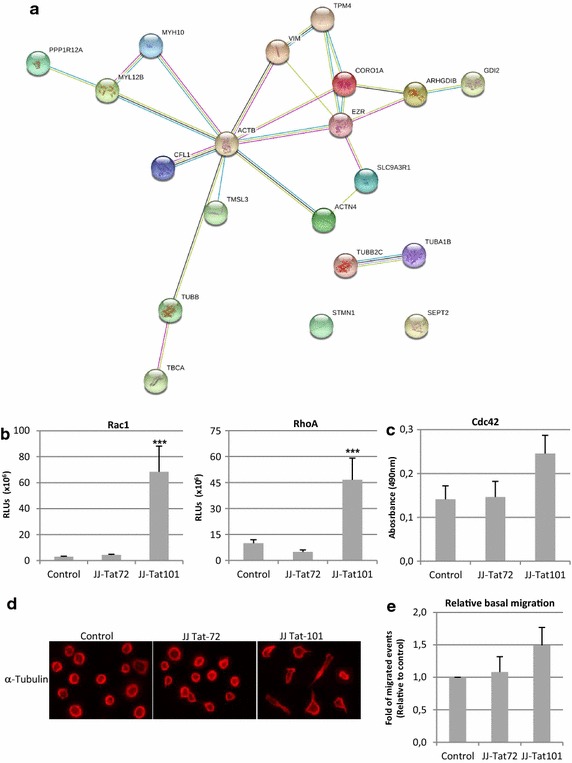
Effect of intracellular Tat expression on cellular polarization and the expression of cytoskeleton-related proteins. **a** Network of predicted interactions between cytoskeletal network proteins deregulated in Jurkat-Tat101 vs control cells (as specified in Table 3). Medium confidence score level was chosen (0.400). Data supporting protein–protein interactions derived from experimental studies (*dark purple lines*), homology (*light purple lines*), databases (*light blue lines*), text mining (*light green lines*), concurrence (*dark blue lines*) and co-expression (*black lines*). Node colour is arbitrary. **b** Rac1 and RhoA GTPases activations were measured using a luminescence-based assay. Data shown are absolute RLUs from three independent experiments. **c** Cdc42 GTPase activation was measured in protein extracts using a colorimetric-based assay. Data shown are absolute absorbance at 490 nm from three independent experiments. **d** Cellular polarization was studied by immunofluorescence in Jurkat-Tat72, Jurkat-Tat101 and control cells adhered to fibronectin using an antibody against α-tubulin and a secondary antibody conjugated with Alexa-555. **e** Analysis of migration capacity in Jurkat-Tat72 and Jurkat-Tat101 in comparison with control cells in the absence of any migratory stimuli. The relative increase of migrated events in comparison with control cells from three independent experiments is shown. Statistical significance was calculated with Kruskal–Wallis test with Dunn’s multiple comparison test (***p < 0.001 vs control)

The interactions between mitochondria and the cytoskeleton influence mitochondrial functions including respiratory activity, fusion/fission processes and ROS biogenesis [36, 37]. Therefore, the expression of cytoskeleton-related proteins was analyzed by LC–MS/MS and predicted protein interactions were analyzed by STRING database (Fig. 7a). Jurkat-Tat101 cells, and to a lesser extent Jurkat-Tat72 cells, showed the deregulation of 20 cytoskeleton-related proteins (Table 3). Only five deregulated proteins were tubulin-related. Fifteen actin-related proteins were altered in Jurkat-Tat101 cells. Three of these proteins were involved in small-GTPase signal transduction, including Rho GDP-dissociation inhibitor 2 (ARHGDIB) and GDP dissociation inhibitor beta protein (GDI2), which were −10.0- and +2.0-fold deregulated. Furthermore, the activation of RhoA and Rac1 GTPase increased 22.38- and 4.67-fold in Jurkat-Tat101 (*p* < 0.005) but not in Jurkat-Tat72 cells (Fig. 7b), in comparison to control cells. Non-significant differences were found in the activation of Cdc42 (Fig. 7c). Finally, cells were stained with an antibody against tubulin to elucidate the cellular shape. Most Jurkat-Tat101 cells showed polarized shape, whereas non-polarized and typically round-shape lymphocytes were observed in Jurkat-Tat72 and control cells (Fig. 7d). The analysis of migration ability showed that Jurkat-Tat101 cells migrated spontaneously 1.5-fold more than Jurkat-Tat72 and control cells (Fig. 7e).

**Table 3:** Selection of proteins deregulated at least ±2.0-fold in Jurkat-Tat101 versus control cells

Gene	Protein	Tat72 vs TetOff	Tat101 vs TetOff	Biological process	Peptide sequence	X-corr
CORO1A	Coronin-1A	9.3	36.8	Actin cytoskeleton organization	KLQATVQELQK	2.93
MYL12B	Myosin regulatory light chain 12B	3.0	13.2	Regulation of cell shape	ELLTTMGDRFTDEEVDELYR GNFNYIEFTR	1.75 2.60
EZR	Ezrin	11.2	11.9	Actin filament bundle assembly	QLLTLSSELSQAR QLLTLSSELSQARDENKR	1.79 2.06
SEPT2	Septin-2	0.0	5.2	Actin cytoskeleton organization	TMLITHMQDLQEVTQDLHYENFR	2.29
CFL1	Cofilin-1	0.9	5.2	small GTPase signal transduction	LGGSAVISLEGKPL	2.33
ACTN4	Actinin-alpha-4	0.0	4.4	Actin filament bundle assembly	MAPYQGPDAVPGALDYK GISQEQMQEFR	2.11 2.11
TUBB2C	Tubulin beta-2C	6.4	4.4	Microtubule organization	INVYYNEATGGK	1.75
SLC9A3R1	Na/H exchange regulatory cofactor (NHE-RF1)	3.1	3.5	Actin cytoskeleton organization	SVDPDSPAEASGLR SVDPDSPAEASGLRAQDR	1.95 1.33
PPP1R12A	Protein phosphatase 1 regulatory subunit 12A	5.2	2.8	Actin cytoskeleton organization	NKETLIIEPEKNASR	2.12
TPM4	Tropomyosin alpha-4 chain	9.9	2.2	Actin cytoskeleton organization	KIQALQQQADEAEDR	1.65 2.13
TBCA	Tubulin-specific chaperone A	2.1	2.2	Tubulin assembly	LVLDSVKLEA RLEAAYLDLQR	1.78 1.84
VIM	Vimentin	3.9	2.2	Cytoskeleton organization	FADLSEAANR	1.75
GDI2	Rab GDP dissociation inhibitor beta	0.0	2.0	small GTPase signal transduction	MTGSEFDFEEMKR	1.78
TUBA1B	Tubulin alpha-1B	3.7	−3.4	Microtubule cytoskeleton organization	TIGGGDDSFNTFFSETGAGK DVNAAIATIK	1.91 2.00
TUBB	Tubulin beta	−1.3	−6.9	Microtubule cytoskeleton organization	FWEVISDEHGIDPTGTYHGDSDLQLDR AILVDLEPGTMDSVR EVDEQMLNVQNK MSMKEVDEQMLNVQNK	2.16 2.20 2.74 4.08
MYH10	Myosin-10	−2.3	−8.5	Actin filament-based movement	ELDDATEANEGLSREVSTLKNR QLEEAEEEATRANASR ELDDATEANEGLSR	1.99 2.38 2.78
ARHGDIB	Rho GDP-dissociation inhibitor 2	−2.6	−10.0	small GTPase signal transduction	TLLGDGPVVTDPKAPNVVVTR	1.97
STMN1	Stathmin	−28.5	−30.5	Microtubule depolymerization	RAsGQAFELILsPR	2.19
TMSL3	Thymosin beta-4-like protein 3	−28.8	−33.4	Actin cytoskeleton organization	NPLPSKETIEQEKQAGES NPLPSKETIEQEK TETQEKNPLPSKETIEQEK	1.80 2.31 2.65
ACTB	Actin cytoplasmic	−15.0	−43.7	Actin cytoskeleton organization	DSYVGDEAQSK DLYANTVLSGGTTMYPGIADR DSYVGDEAQSKR HQGVMVGMGQK AVFPSIVGRPR TTGIVMDSGDGVTHTVPIYEGYALPHAI QEYDESGPSIVHR HQGVMVGMGQKDSYVGDEAQSK IWHHTFYNELR MQKEITALAPSTMK	1.83 1.87 1.88 1.89 1.98 2.15 2.37 2.81 3.31 3.52

Only proteins related to actin and tubulin cytoskeleton networks were selected. All peptides selected showed 95 % probability for protein expression and a minimum X-corr of 1.75

### Intracellular Tat101 increased mitochondrial amount and polarized its distribution

**Fig. 8:**
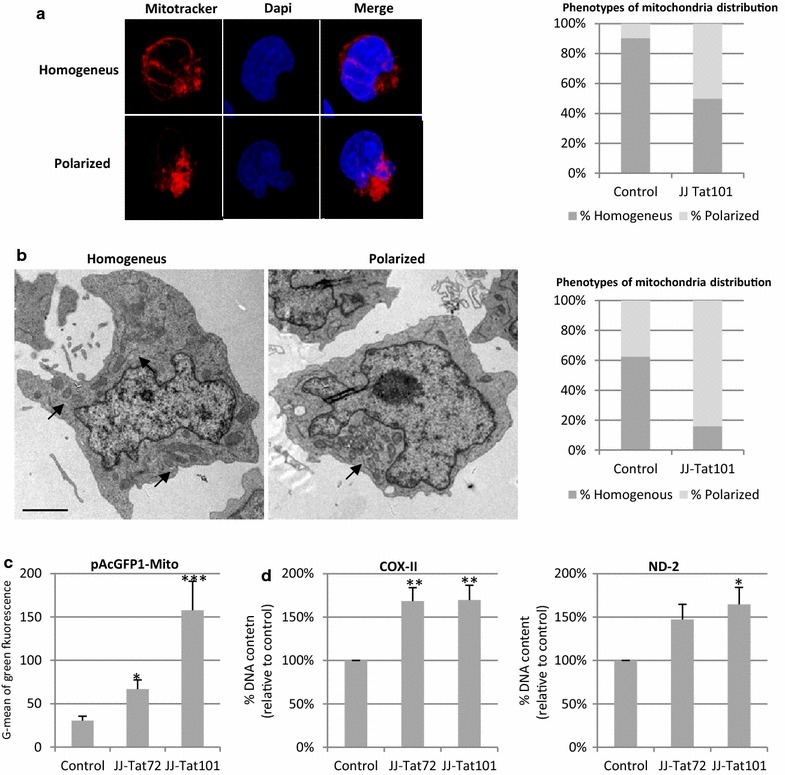
Effect of intracellular Tat expression on mitochondrial mass and its subcellular distribution. **a** Mitochondria distribution within Jurkat-101 vs control cells was analyzed by microscopy after staining with Mitotracker probe. The nucleus was stained with DAPI. Representative images of a cell with homogeneous and polarized mitochondrial mass distribution are shown. The *graph* includes the percentage of each phenotype in Jurkat-Tat101 and control cells. **b** Mitochondria network distribution was also analyzed by electron microscopy using ultrathin sections. Representative images from control and Jurkat-Tat101 cells showing the homogeneous and polarized mitochondrial network distribution are shown. *Scale bar* corresponds to 200 nm. The *graph* shows the percentage of each phenotype in Jurkat-Tat101 and control cells. **c** Expression of the nuclear-encoded VIII sunit of the cytochrome *c* oxidase was analyzed by flow cytometry in Jurkat-Tat72, Jurkat-Tat101 and control cells transfected with a vector coding for this protein bound to GFP (pAcGFP1-Mito). *Graph* shows G-mean of green fluorescence intensity of the living cell population from three independent experiments. Statistical significance was calculated with Kruskal–Wallis test with Dunn’s multiple comparison test (*p < 0.05 and ***p < 0.001). **d** qPCR measurement of mitochondria DNA matching with regions coding for COX-II and MTND-2 genes. Nuclear-encoded DNA S18 was used as house-keeping gene. Data shown are media and SEM from three independent experiments. Statistical significance was calculated with Kruskal–Wallis test with Dunn’s multiple comparison test (*p < 0.05 and **p < 0.01)

Because mitochondria are transported to the uropod to ensure the high ATP demand required for lymphocyte migration [38–40] and Jurkat-Tat101 cells showed an overall cellular polarized shape, the distribution of the mitochondria network was studied. The mitochondria network of Jurkat-Tat101 and control cells was analyzed after incubating with Mitotracker and DAPI. Two different phenotypes, which included a homogeneous and a polarized distribution of the mitochondria network, were identified (Fig. 8a). The overall presence of the polarized phenotype increased up to 50 % in Jurkat-Tat101 cells whereas in control cells reached 9.5 %. These phenotypes corresponding to homogeneous and polarized distribution of the mitochondria network were also identified in Jurkat-Tat101 cells by electron microscopy (Fig. 8b). The re-distribution of mitochondria at one edge of the cell was detected in 84.0 % of Jurkat-Tat101 cells but only in 36 % of control cells. However, ultrastructure changes were not detected and in both types of cells the overall size and shape of mitochondria were similar, showing mitochondria with normal crista, enlarged shape and double organelle membrane.

Then, average amount of mitochondria was quantified in Tat expressing cells. Jurkat-Tat101 and Jurkat-Tat72 cells were transfected with pAcGFP1-Mito vector that encodes a green fluorescent protein (GFP) fused with a precursor of the cytochrome c oxidase VIII subunit in order to quantify the average amount of mitochondria. As mitochondrial pre-proteins are rapidly degraded in the cytoplasm by proteasomes when they are not properly imported into the mitochondria, GFP from pAcGFP1-Mito vector only occurs when the protein targets the mitochondria [41]. G-mean of green fluorescence intensity of the living cell population increased 5.17-fold in Jurkat-Tat101 cells but only 2.35-fold in Jurkat-Tat72 cells, as compared to control cells (*p* < 0.005) (Fig. 8c). The levels of two mtDNA-segments that match with the area of COX-II and MTND-2 genes were measured by qPCR. The amount of mtDNA COX-II and MTND-2 regions increased 1.7-fold in Jurkat-Tat101 cells (*p* < 0.005), while it only occurred in COX-II mtDNA fragment in Jurkat-Tat72 cells (*p* < *0.05*) (Fig. 8d). This result confirms data from Fig. 2a showing enhanced mitochondria content in Jurkat-Tat cells.

### PBLs expressing Tat-101 showed decreased synthesis of ATP and enhanced levels of lactate

**Fig. 9:**
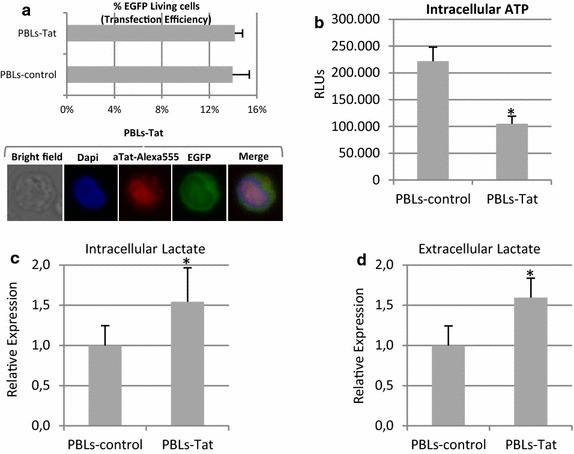
Intracellular ATP production and intracellular lactate levels and release in PBLs expressing Tat101. Resting PBLs were transiently transfected with vectors CMV-Tat101 or pcDNA3, as negative control, along with pEGFP expression vector, as control of transfection efficiency. **a** Flow cytometry quantification of the percentage of living cells expressing EGFP was used to analyze transfection efficiency. Intracellular expression of Tat and nuclear subcellular localization were confirmed by immunofluorescence using a monoclonal antibody against Tat and a secondary antibody conjugated to Alexa 546. DAPI was used for nuclear staining. **b** ATP production was measured by chemiluminescence using a commercial assay. Data shown are RLUs mean and SEM of concentration from five independent experiments. **c** Relative intracellular lactate production measured in PBLs expressing Tat versus control PBLs. Data shown are relative mean and SEM of concentration from five independent experiments. **d** Relative release of lactate to the culture medium measured in the same PBLs expressing Tat used to measure intracellular lactate. Statistical significance was calculated by Mann–Whitney test (*p < 0.05)

PBLs are a more physiological cellular system than T CD4+ Jurkat lymphocytes. Representative data were confirmed using peripheral blood lymphocytes (PBLs) from healthy donors that were transfected with a vector expressing Tat101 protein or with pcDNA3 as negative control. pEGFP vector was co-transfected to measure the transfection efficiency, which was 15 % in all cases (Fig. 9a). Nuclear localization of intracellular Tat was confirmed by immunofluorescence. The intracellular levels of ATP were significantly 2.12-fold reduced in PBLs transiently expressing intracellular full-length Tat (*p* < 0.05) (Fig. 9b).

Relative data showing lactate concentrations in PBLs expressing Tat after normalization with lactate concentrations in control PBLs from the same donor are shown. In PBLs transiently expressing Tat101, intracellular and extracellular lactate levels increased 1.54- and 1.60-fold (*p* < 0.05), respectively (Fig. 9c, d). Relative instead of absolute experimental data are shown because both intracellular and extracellular lactate concentrations varied among independent donors, but the same tendency was observed in each experiment. These results support our previous findings in Jurkat-Tat101 cells shown in Fig. 2.

### HIV-infected T lymphocytes shared some mitochondrial alterations with Tat expressing cells

**Fig. 10:**
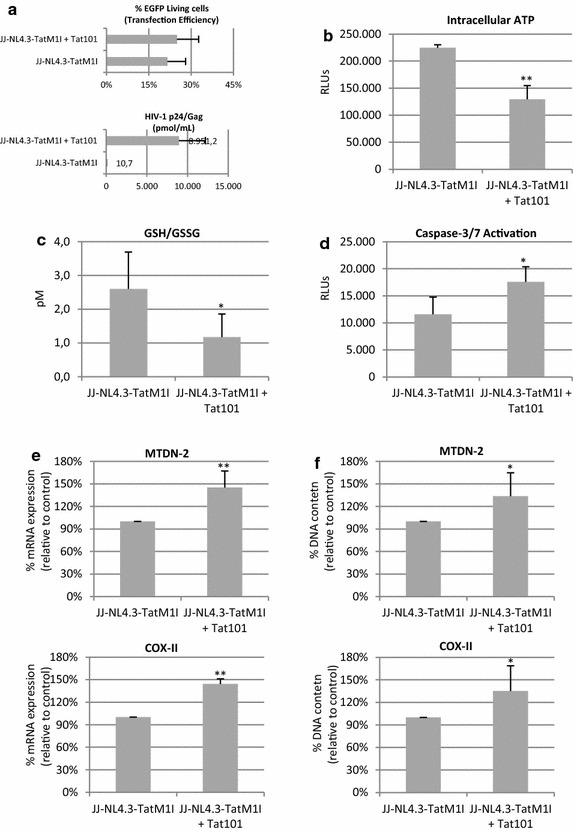
Intracellular ATP levels, GSH/GSSG ratio, caspase-3/7 activation, mtDNA transcription and mitochondrial content in HIV-1 infected T lymphocytes. PBLs were infected pNL4.3-TatM1I vector along with pcDNA3 or with pCMV-Tat101 vector. pEGFP was co-transfetion as a control of transfection efficiency. **a** Flow cytometry quantification of the percentage of living cells expressing EGFP was used to analyze transfection efficiency (*upper panel*). Quantification of p24/Gag in culture supernatant was used as control of infection (*lower panel*). **b** Intracellular ATP production. **c** Intracellular concentration of GSH/GSSG. **d** Caspase-3/7 activation. Intracellular ATP, GSH/GSSG ratio and caspase-3/7 activation were measured by chemiluminescence using commercial assays. **e** qPCR analysis of mRNAs levels from the mtDNA-encoded genes MTND-2 and COX-II. mRNA levels of nucelar-encoded S18 expression were used as house-keeping gene. **f** qPCR analysis of mitochondria DNA matching with regions coding for COX-II and MTND-2 genes. Nuclear-encoded DNA S18 was used as house-keeping gene. Data shown are mean and SEM from three independent experiments. Statistical significance was calculated by Mann–Whitney test or Kolmogorov–Smirnov test (*p < 0.05, **p < 0.01)

The importance of Tat in modifying mitochondrial main functions was evaluated in the context of viral replication. Jurkat cells were infected by electroporation with the Tat-defective viral genome pNL4.3-TatM1I [15] along with pTat101 or pcDNA3 as control vector. pEGFP vector was co-transfected to measure the transfection efficiency, which was 21 and 25 % in control cells and in HIV-infected cells, respectively (Fig. 10a, upper panel). HIV-1 replication was assessed by quantifying p24/Gag levels in the culture supernatants (Fig. 10a, lower panel). The levels of p24/Gag increased 652.9-fold in transfected with NL4.3-TatM1I and expressing Tat101 versus control cells infected only with defective NL4.3-TatM1I. Control cells expressed similar levels of p24/Gag than cells transfected with pcDNA3 alone (data not shown). As HIV-1 replication occurred from vector expression, expression levels of Tat in pNL4.3-TatM1I transfected Jurkat cells were compared to Tat levels in Jurkat cells infected with HIV-1. Immunoblotting analysis showed that Tat expression levels were 2.0-fold increased in Jurkat cells transfected with pNL4.3-TatM1I along with pCMV-Tat101 vectors in comparison to Tat levels in Jurkat cells infected with NL4.3 virions (Additional file 1: Fig. S1a). Accordingly, HIV-1 replication, measured by quantifying p24/Gag levels in the culture supernatant, was also 3.3-fold enhanced in transfected *versus* infected cells (Additional file 1: Fig. S1b). These data suggest that Tat expression from transfection or infection may be equivalent in Jurkat cells. Neither Tat expression nor HIV-1 replication were found in non-infected Jurkat cells or in Jurkat cells transfected with pNL4.3-TatM1I along with pcDNA3 (Additional file 1: Fig. S1a, b).

The intracellular levels of ATP were 1.73-fold reduced in HIV-1 infected cells (*p* < 0.001) (Fig. 10b). The GSH/GSSG ratio was significantly 2.22-fold reduced (*p* < 0.05) (Fig. 10c) and caspase-3/7 activation was 1.52-fold enhanced (*p* < 0.05) (Fig. 10d) in HIV-1 infected cells.

Mitochondrial content was measured by quantifying the levels of two mtDNA-segments matching with COX-II and MTND-2 genes and was 1.35-fold enhanced in HIV-infected cells (*p* < 0.05) (Fig. 10f). These data suggest that intracellular Tat101 is responsible for reduced ATP levels, caspase-3/7 activation and enhanced mitochondrial content during HIV-1 infection in T lymphocytes.

## Discussion

Mitochondrial OXPHOS is altered in PBMCs from naïve patients [6, 7], demonstrating that HIV-1 replication per se results in mitochondrial damage independent of ART. In this work, we show that intracellular expression of Tat101 partly leads this process.

The expression of nuclear encoded genes involved in mitochondrial functions was upregulated in Jurkat-Tat101 cells but the transcription of mtDNA genes was dramatically resulting in the impairment of mitochondria functions. This lack of coordination between mtDNA and nuclear genes expression may also result in a defective OXPHOS [42]. Accordingly, complex-I and complex-V were inhibited in Jurkat-Tat101 cells. During HIV-1 infection, the inhibition of complex-I activity reduces the levels of ATP, although ATP synthesis does not stop completely [8]. Furthermore, the reduction of ATP activates a phosphofructokinase that finally increases the concentration of intracellular lactate [43]. Indeed, ATP levels were reduced and subsequently intracellular lactate was accumulated in the cytoplasm of PBLs transiently expressing Tat, CD4+ T cells expressing Tat101 and HIV-1 infected T cells. The modest effect of Tat observed in PBLs was probably due to lower transfection efficiency. It results in the dilution of Tat-mediated effects in the whole population of PBLs, as most of them were not expressing Tat intracellularly.

Lactate can trigger the degradation of IκBα, resulting in the autocrine activation of the transcription factor NF-κB [44], which is essential for HIV-1 replication and gets activated upon intracellular Tat expression [14, 15, 45]. Intracellular lactate levels are secondly regulated by the use of the specific monocarboxylate transporter 1 (MCT1) that allows the diffusion of the lactate out of the cells [46]. Extracellular amounts of lactate did not significantly change in Jurkat-Tat cells, suggesting that high amounts of lactate can be supported without resulting in toxicity. However, enhanced lactate release was found in PBLs from healthy donors expressing Tat protein and in HIV-1 infected cells, reducing the possibility that Tat modifies the MCT1 during HIV-1 infection. Elevated lactate in blood is a noticeable toxic effect of ART and is used to define mitochondrial toxicity in treated HIV-1 patients [47].

Compensatory mechanisms for mitochondria toxicity, including mtDNA increase, have been shown in different subsets of human cells subjected to ART [47–49]. However, PBMCs from naïve patients show mtDNA depletion [6]. Here, we demonstrated that Tat101 increased the mitochondria content in T lymphocytes but not enough to compensate mitochondria impairment. Other discrepancies regarding mtDNA content have been described in adipose tissues from non-treated HIV-1 patients [50, 51], suggesting that the effect of HIV-1 on mitochondria may depend on connected factors that remain to be fully elucidated. Here, an increase of mitochondria content was described in HIV-1 infected cells, contrary to Jurkat-Tat101 cells. This finding suggests that during HIV-1 replication other viral proteins compensate Tat-mediated mitochondrial impairment, although not completely as ATP synthesis was also compromised in HIV-1 infected T cells.

In Jurkat-Tat101 cells, a compensatory mechanism may be changing the cellular preferred metabolism into aerobic glycolysis through a generalized up-regulation of glycolytic enzymes. Indeed, HIV-1 infected cells show a metabolic reprogramming that reduces the energy production from the tricarboxylic acid cycle to aerobic glycolysis [52, 53]. The aerobic glycolysis is a metabolic program, occurring usually in activated lymphocytes [25, 54], in which the lactate is produced in the presence of oxygen, to differentiate it from the anaerobic fermentation of glucose to lactate that arose from faults in respiration [24]. As it was discussed above, lactate production was enhanced in Jurkat-Tat101 cells, PBLs expressing Tat101 and HIV-1 infected cells. Furthermore, our data suggest that the main breakdown point may include the synthesis of pyruvate and the intermediate metabolite glyceraldehyde 3-phosphate (G3P), probably through a dramatic repression of GAPDH. Additionally, intracellular Tat101 enhanced the expression of PKM2 and LDHB, which together increase the conversion of lactate from pyruvate in the cytoplasm. PKM2 is crucial for metabolism shift during HIV-1 pathogenesis, as up-regulation of PKM2 has been observed in different types of HIV-1 infected cells [52, 55, 56]. In contrast to OXPHOS, glycolysis is energetically inefficient, theoretically yielding two molecules of ATP per glucose molecule consumed compared to thirty-six if it is fully oxidized. However, aerobic glycolysis has the energetic advantage of generating ATP at higher speed than OXPHOS [57]. Therefore, activated lymphocytes do not activate aerobic glycolysis only because their capacity for OXPHOS is saturated but also to provide sufficient ATP to support the cellular growth and proliferation [58–60]. In summary, aerobic glycolysis may be activated in Jurkat-Tat101 cells as a complementary source of ATP production.

Increased production of ROS due to diminished complex I activity and subsequent reduction in ATP levels has been extensively described in infected CD4+ T cells [8]. Indeed, extracellular Tat is known to increased generation of ROS and caspase-3/7 activation in the intestinal mucosa of HIV-infected patients [20]. In this work, it is shown that intracellular Tat101 increased ROS production and apoptosis induction, and both processes were coupled. However the GSH/GSSG ratio was balanced in Jurkat-Tat101 cells despite of the chronic oxidative stress detected in HIV-1 infected lymphocytes. Therefore, our data cannot point out a role of intracellular Tat expression in the overall glutathione imbalanced showed in PBLs from HIV-1 infected patients. Because of the experimental systems used in this work, we cannot rule out the role of other viral proteins in the induction of oxidative stress and mitochondrial damage in infected T cells. In particular, extracellular Tat and other viral proteins as extracellular Vpr are able to reduce GSH/GSSG ratio [20, 61], and may gain value during HIV-1 infection in vivo. Although chronic oxidative stress is detected in HIV patients [62, 63], most in vivo studies are focused on patients under ART, where viral load is not detectable [64–67] and treatment enhances the synergy of HIV and oxidative stress [68]. This fact together with the low number of productively infected lymphocytes in peripheral blood HIV-1 in vivo infection [69, 70] suggest that not only HIV-1 replication itself but additional factors may contribute to oxidative stress as part of HIV-1 disease pathogenesis. This phenomenon may be related to the chronic immune activation and senescence produced by increased microbial translocation and persistent viremia or secondary to the increased rate of opportunistic infections as it has been described [71–73].

ROS levels are responsible for the profound reorganization of mitochondria-cytoskeletal interactions found in HIV-1 patients [7]. Furthermore, ROS lead to the appearance of dense stress fibers, required for intercellular contacts and migration [74]. In line with it, the mitochondria network of Jurkat-Tat101 cells was localized at one edge of the lymphocyte, reflecting the acquisition of a polarized shaped and probably as a host defense response to supply enough energy to the migration process. Because mitochondria are transported to the uropod along microtubules [38], it seems that microtubule dynamics are persevered in Jurkat-Tat cells for mitochondria trafficking and delocalization at the uropod. Furthermore, profound actin cytoskeleton changes may also be responsible for Tat-mediated mitochondria delocalization. For instance, intracellular Tat101 activated RhoA and Rac1, actin remodeling GTPases that control mitochondria trafficking [75], probably through the depletion of ARHGDIB, a protein that promotes the misfolding and degradation of the cytosolic pool of Rho GTPases together with the activation of the remaining membrane bound pool [76, 77]. Accordingly, mitochondria proteome from HIV-infected patients are enriched in actin proteins [7], including the GTPase Rab1, which regulates organelle tethering through the enhancement of Rab GDP dissociation inhibitor beta protein (GDI2) [78], a modulator of Rab recycling that is up-regulated in Jurkat-Tat cells. Eventually, mitochondria accumulation also requires an unperturbed balance between fusion and fission processes [38], which might occur in Jurkat-Tat101 cells as the expression of pro-fusion *MFN2* and pro-fission *DNM1L* genes were not deregulated.

It is to notice that Tat is indispensable for HIV-1 replication [13, 79, 80] and it is not packaged inside the virions [81]. Accordingly, NL4.3-TatM1I strain lacking Tat protein did not yield productive infection. Therefore only the transfection approach based on co-expressing NL4.3-TatM1I strain and Tat101 allowed a proper assessment of the impact of Tat on the different mitochondrial parameters during HIV-1 replication. Therefore, results from Jurkat expressing only intracellular Tat do not always correlate with data from infection, where other viral proteins exert compensatory mechanisms to success viral replication.

## Conclusions

This work shows the influence of intracellular HIV-1 Tat protein on mitochondrial overall functions in Jurkat cells and PBLs. Tat101 polarized the mitochondria distribution and increased the mitochondrial content, probably to compensate mtDNA transcription impairment and low ATP production. Tat101 also enhanced the expression of metabolism-related proteins and mitochondrial-related genes. The presence of Tat second exon increased these effects, confirming that full-length Tat mostly regulated the non-transcriptional functions of the protein. The intracellular expression of Tat may be responsible for the mitochondrial dysfunctions found in HIV-1 infected patients as for instance OXPHOS impairment and increased mtDNA content and therefore Tat may enhance the susceptibility to mitochondrial toxicity during ART.

## Methods

### Cells

Jurkat TetOff cell line was purchased from BD Biosciences Clontech (Mountain View, CA, USA). The high-level gene expression TetOff system [82], which keeps the expression of the gene cloned in pTRE2hyg vector continually turned on. Unlike other inducible mammalian expression systems, gene regulation in the Tet systems is highly specific, so the interpretation of results would not be hindered by pleiotropic effects or nonspecific induction (Yin et al. 1996). The vector pTRE2hyg-Tat101 expressed a complete HIV-1 tat gene (aa 1–101), obtained from pCMV-Tat101 vector [83], after cloning in pTRE2hyg vector (Clontech, BD Tet-Off gene expression System, BD Biosciences). Complementary DNA (cDNA) from tat first exon (1–219nt; 1–72aa) was obtained from pCMV-Tat101 vector using specific oligonucleotides to introduce a stop codon at residue 73, and then cloned in pTRE2hyg vector. Jurkat TetOff was transfected by electroporation with pTRE2hyg-Tat72 or pTRE2hyg-Tat101 vectors and these cell lines were stabilized with hygromycin B as previously described [14, 16]. The empty pTRE2hyg vector was transfected and stabilized in the Jurkat TetOff cell line into obtained negative control cells. Jurkat-Tat101 and Jurkat-Tat72 cells are not clone populations but a mixed population in which more than 75 % of the cells express high intracellular amounts of Tat101 (full-length) or Tat72 (first exon) protein which were equivalent to Tat levels detected in T lymphocytes infected with the HIV-1 infectious clone NL4.3wt [16]. All Jurkat cells were cultured in RPMI 1640 medium (Biowhittaker, Walkersville, MD, USA) with 10 % fetal calf serum (PAN Biotech GmbH, Aidenbach, Germany), 2 mM l-glutamine, 100 μg/ml streptomycin and 100 U/ml penicillin (Lonza, Basel, Switzerland), 300 μg/ml geneticin and 300 μg/ml hygromycin B (BD Biosciences Clontech), at 37 °C and 5 % CO_2_. Serum depletion was performed 3 h before each experiment. PBLs were isolated from blood of healthy donors by centrifugation through a Ficoll-Hypaque gradient (Pharmacia Corporation, North Peapack, NJ, USA) and cultured in RPMI 1640 medium supplemented with 10 % (v/v) fetal calf serum (FCS), 2 mM l-glutamine, 100 μg/ml streptomycin, 100 U/ml penicillin (Biowhittaker, Walkersville, MD, USA).

### Antibodies, reagents and vectors

Anti-tubulin, MitoTracker CMxRos probe (0.2 μM) and 4′,6-diamidino-2-phenylindole (DAPI) were purchase from Sigma-Aldrich. Antibody against HIV-1 Tat was obtained from Advanced Biotechnologies Inc. (Columbia, MD, USA). pCMV-Tat101 vector expresses HIV-1 Tat101 protein [84]. pAcGFP1-Mito vector (BD Biosciences, Erembodegem, Belgium) encodes the GFP derived from *Aequorea coerulescens* fused at its N-terminus with a precursor of the cytochrome c oxidase VIII subunit [41].

### Proteome analysis and criteria for protein identification

Proteome analysis was performed as previously described [15]. Briefly, 200 μg of trypsin-digested proteins were loaded into the LC–MS/MS system and analyzed using a C-18 reversed phase nano-column (Thermo-Fisher, San Jose, CA, USA). Real-time ionization and peptide fragmentation was performed on an orbital ion trap mass spectrometer (LTQ Orbitrap XL, Thermo Fisher Scientific, San Jose, CA, USA) [85]. For peptide database searching, tandem mass spectra were analysed using Sequest (Thermo Fisher Scientific; version 1.3.0.339) and X! Tandem (http://www.thegpm.org; version CYCLONE). Sequest and X! Tandem were searched with a fragment ion mass tolerance of 30 PPM and a parent ion tolerance of 15 PPM. Scaffold 3.0 (Proteome Software Inc., Portland, OR, USA) was used to validate peptide identifications. Only peptide established at a probability greater than 95 % and with XCorr-score values above 1.75 were accepted. Proteins related to mitochondria and cytoskeleton that changed at least ±2.0-fold in Jurkat-Tat101 were selected and subjected to analysis with STRING 9.0 database (http://string-db.org/) [86].

### PCR and RT-PCR assays

DNA and total RNA was isolated with QiAmp DNA blood mini kit and RNeasy Mini kit (Qiagen, Barcelona, Spain), respectively. cDNA was synthesized using GoScript Reverse Transcription System (Promega, WI, USA). Specific primers are described in Additional file 2: Table S1. The expressions Ribosomal protein S18 (S18) was used as housekeeping gene. The PCR amplification was performed in a StepOnePlus™ Real-Time PCR Systems (Applied Biosystems, CA) using SYBR Green. Data analysis was performed with 7500 software v2.0.6 and Ct values were normalized according to S18 amplification and analyzed with the formula 2^−ΔΔCt^.

### Expression of nuclear-encoded genes related to mitochondria

RT^2^ Profiler™ PCR Array Human Mitochondria (Qiagen) is a RT-PCR based array and was used to studied the expression of 84 mitochondrial related genes which are nuclear encoded. The array also included five housekeeping genes and internal controls for genomic DNA amplification, reverse transcription and positive PCR controls. Genomic RNA elimination and reserve transcription was performed using 5 μg of total RNA of Jurkat-control and Jurkat-Tat101 cells, following manufacturers’ instructions. The PCR amplification was performed in a StepOnePlus™ Real-Time PCR Systems (Applied Biosystems) using SYBR Green. Data analysis was performed accordingly to the instructions detailed in RT^2^ Profiler™ PCR Array Human Analysis Manual. C_T_ higher than 30 cycles were rejected for further analysis. C_T_ values were normalized according to the amplification of five housekeeping genes included in the array amplification and analyzed with the formula 2^−ΔΔCt^.

### Activity of citrate synthase and OXPHOS complexes I and V

The enzymatic activity of citrate synthase (EC 2.3.3.1) was measured with Citrate Synthase Assay Kit (Sigma-Aldrich). Briefly, 1 × 10^7^ cells were lysed with 125 μl of CelLytic M cell Lysis Reagent and 10 μl of this lysate was incubated with 200 μl of a reaction mix containing acetyl-coA, DTNB and oxaloacetic acid. Changes in the absorbance at 412 nm were followed in a microplate reader Sunrise (Tecan Group Ltd., Männedorf, Switzerland) to calculate the units (μmole/ml/min) of citrate synthase accordingly to the manufacturer’s instructions. The activities of complex I (NADH dehydrogenase or ubiquinone oxidoreductase, EC 1.6.5.3) and complex V (F1F0 ATPase or ATP synthase, EC 3.6.3.14) were measured using complex I Enzyme Activity Microplate Assay Kit (Abcam) and ATP synthase (complex V) Human Profiling ELISA Kit (Abcam), respectively, according to manufacturer’s instructions. Complex I assay is based on the oxidation of nicotinamide adenine dinucleotide (NADH) to NAD+ and the simultaneous reduction of a dye leading to an increase of absorbance at 450 nm, which was measured in a microplate reader Sunrise. Briefly, protein from 6 × 10^6^ cells were extracted by diluting 1/10 the extraction buffer. A total of 200 μg of protein from supernatants in 50 μl of final volume was added in each well and incubated for 3 h at room temperature before accurate washing and adding 200 μl of assay solution, containing 40 mM NADH. The absorbance was measured at 450 nm at approximately 30 s’s intervals for 30 min. To quantify complex-V activity, a total of 200 μg of protein extracted 6 × 10^6^ cells was added in each well which is coated with a capture antibody specific for human ATP synthase. After 2 h incubation at room temperature and continuous shaking at 300 rpm, wells were washed and further incubated with an anti- ATP synthase detector antibody and then with a specific HRP-conjugated antibody. The TMB substrate solution and hydrochloric acid used as stopping solution were added to each well and the color intensity was measured at 450 nm using a microplate reader Sunrise. To assess OXPHOS complex I and complex V activities, absorbance values were normalized accordingly to mitochondria content using citrate synthase levels.

### ATP and lactate concentrations

Intracellular levels of ATP were determined with CellTiter-Glo^®^ 9 Luminescent Cell Viability Assay (Promega), following the manufacturer’s instructions. Briefly, 1 × 10^5^ cells were incubated for 10 min at room temperature in CellTiter-Glo^®^ Reagent, which contained lysis buffer and thermostable luciferase and the luminescent signal was analyzed in an Orion Microplate Luminometer with Simplicity software (Berthold Detection Systems, Oak Ridge, TN, USA). Intracellular and extracellular levels of l-(+)-Lactate were measured in cell lysates or supernatants using Lactate Assay Kit II (Sigma-Aldrich) accordingly to manufacturer’s instructions. Briefly, 1 × 10^6^ cells were lysed with 4 volumes of lysis buffer or pelleted to collect the subsequent supernatant and 50 μl of each sample was incubated with the commercial enzyme during 30 min at room temperature. The absorbance at 450 nm, which was proportional to the lactate concentration, was determined using in a microplate reader Sunrise.

### Intracellular ROS and glutathione levels

Living Jurkat-Tat cells were adhered onto fibronectin-coated plates and stained with DCF-DA 2′,7′-(5 μM) (Sigma-Aldrich), which after oxidation is converted into highly fluorescent DCF (2′7′-dichlorofluorescein) [26]. Images were obtained with a Leica DMI 4000B Inverted Microscope (Leica Microsistemas, Barcelona, Spain). Cells from a total of 15 fields from three independent experiments were analyzed. Fluorescence from cells stained with DCF-DA-FITC was also measured by FACScalibur Flow Cytometer (BD Biosciences Clontech) using FL-1 channel and data were analysed by CellQuest software. Intracellular levels of reduced glutathione (GSH) and oxidized glutathione (GSSG) were measured using the luminescence-based GSH/GSSG-Glo Assay (Promega). The luminescent signal (relative light units, RLUs) was quantified in an Orion Microplate Luminometer with Simplicity software and RLUs were converted into concentration (pM) using the glutathione standard curve included in the assay.

### Apoptosis assays

Living cells were adhered on PolyPrep slides, fixed with 2 %-PFA and treated with Dapi (4′,6-diamidino-2-phenylindole) (Sigma-Aldrich) for staining the nuclei. Images were obtained with a Leica DMI 4000B Inverted Microscope (Leica Microsistemas, Barcelona, Spain). The percentage of apoptotic events was calculated by acquiring 60 fields from three independent experiments—containing an average number of cells close to 40—and considering the total number of cells. Activation of casapse-3 was measured in non-treated cells with CaspaseGlo^®^3/7 systems (Promega). The luminescent signal (relative light units, RLUs), which was directly proportional to caspase activation, was quantified in an Orion Microplate Luminometer with Simplicity software (Berthold Detection Systems). Cells were stained with Annexin-V-PE (Immunostep, Salamanca, Spain) to measure apoptosis commitment. Cells were co-stained together with DCF-DAC and Annexin-V-PE to measure the co-localization of apoptosis and ROS production. Fluorescence was measured by FACScalibur Flow Cytometer (BD Biosciences Clontech) and data were analyzed by CellQuest software.

### Immunofluorescence and electron microscopy assays

Living cells were stained with MitoTracker Red CMxRos Mitochondrial (0.2 μM) and then adhered on PolyPrep slides (Sigma-Aldrich) and fixed with 2 % paraformaldehyde (PFA) in PBS1x. To study cellular polarization, cells were adhered on fibronectin-coated slides, fixed with 2 %-PFA and stained with anti-tubulin by immunofluorescence assay previously described [14]. Nuclei were stained using 4′,6-diamidino-2-phenylindole (Dapi) from Sigma-Aldrich. Cells from a total of 15 fields from three independent experiments were analyzed. Images were obtained with a Leica DMI 4000B Inverted Microscope (Leica Microsistemas, Barcelona, Spain). For electron microscopy ultrastructural analysis, cells were fixed with a mixture of 2 % glutaraldehyde and 4 % PFA 0.1 M phosphate buffer (pH 7.4) for 2 h at 4 °C, washed three times in phosphate buffer; and postfixation was performed with a 1 % osmium tetroxide and 1 % potassium ferricyanide and 0.15 % tannic acid. Samples were treated with 2 % uranyl acetate and dehydrated in increasing concentrations of ethanol (50, 75, 90, 95, and 100 %). Finally, infiltration in epoxy-resin was done using increasing concentrations of resin (25, 50, 75 and 100 %) and polymerization was performed at 60 °C. Ultrathin sections of the samples were stained with saturated uranyl acetate and 2 % lead citrate following standard procedures and sections were imaged with a Tecnai 12 FEI microscope (FEI, Hillsboro, Oregon, USA) operated at 120 kV.

### Activation of small GTPases and cellular migration

Rac1 and RhoA GTPase activation was measured using G-LISATM Rac1 activation Assay Biochem Kit™ and G-LISA™ RhoA activation Assay Biochem KitTM (Cystoskeleton, Denver, CO, USA), which are luminescence based. Cdc42 GTPase activation was measured with the colorimetric assay G-LISATM Rac1 activation Assay Biochem Kit™ (Cystoskeleton). Migration of living cells was allowed for 2 h at 37 °C with 5 % CO_2_ in transwell plates with 5 μm-pore filters (Costar, Cambridge, MA, USA) and quantified by flow cytometry.

### HIV-1 infection

Jurkat cells were in vitro infected by electroporation. pNL4.3-TatM1I vector was co-transfected along with pCMV-Tat101 in a proportion 2:1 as previously described [15]. pNL4.3-TatM1I vector is similar to pNL4.3 wildtype but contains a point mutation in the start codon of the tat gene, and therefore it is not able to infect productively [15]. pCMV-Tat101 vector expresses HIV-1 Tat101 wild type protein [84]. pNL4.3-TatM1I vector co-transfected with pcDNA3 vector was used as negative control. pEGFP vector (BD Biosciences Clontech) was used as control of transfection efficiency. The production of infectious HIV-1 progeny was confirmed by quantifying the levels of p24/Gag in the culture supernatant 48 h post-transfection.

### Quantification of Tat levels in HIV-1 infected cells

Briefly, infectious supernatants were obtained from calcium phosphate transfection of HEK293T cells with pNL4.3-wt plasmid. Jurkat cells were infected with 10 ng of p24/Gag equivalent of NL4.3 strain per million cell by spinoculation during 30 min at gently rotation and room temperature. Non-infected Jurkat cells were used as negative control. Jurkat cells were also infected by electroporation. pNL4.3-TatM1I vector was co-transfected along with pCMV-Tat101 or with pcDNA3 vector as negative control. pEGFP vector was used as control of transfection efficiency. Whole protein extracts were obtained 5 days post-infection and 100 ug were fractionated by sodium dodecyl sulfate–polyacrilamide gel electrophoresis (SDS–PAGE). Expression levels of Tat were analyzed by immunobloting analysis (western-blot) using mouse monoclonal antibody to HIV-1 Tat regulatory protein (aa 1–16) (Biotechnologies, ABI, Columbia). Images were acquired in a BioRad Geldoc 2000 and densitometry was performed with Quantity One software (BioRad Laboratories, Madrid, Spain) with Quantity One software. The relative ratio of the optical density units corresponding to each sample was calculated regarding the internal control of each lane after subtracting background noise.

### Statistical analysis

Statistical analysis was performed using Graph Pad Prism 5.0 (San Diego, CA, USA). Comparisons among control, Jurkat-Tat72 and Jurkat-Tat101 cells were made using non-parametric Kruskal–Wallis test with Dunn’s Multiple Comparison post hoc analysis. Non-parametric Mann–Whitney test was used to compare PBLs expressing or not Tat101 and HIV-1 infected cells versus control. Non-parametric Kolmogorov–Smirnov test was used to compared DNA and RNA PCRs data in HIV-1 infected cells versus control, as normalized data yielded the same value in control cells. The *pvalues* (*p*) <0.05 were considered statistically significant.

## Additional files

Additional file 1: Figure S1. Tat levels during HIV-1 infection in T cells.
Additional file 2: Table S1. List of oligonucleotides designed for mtDNA amplification.
