# Supplementary material for: Intracellular expression of Tat alters mitochondrial functions in T cells: a potential mechanism to understand mitochondrial damage during HIV-1 replication
Source: Retrovirology. 2015 Sep 16;12:78. doi: 10.1186/s12977-015-0203-3 (PMC4571071; doi:10.1186/s12977-015-0203-3)
Supplement: Supplementary file 1 — Additional file 1: Figure S1. Tat levels during HIV-1 infection in T cells. [file 12977_2015_203_MOESM1_ESM.ppt]

## Slide 1
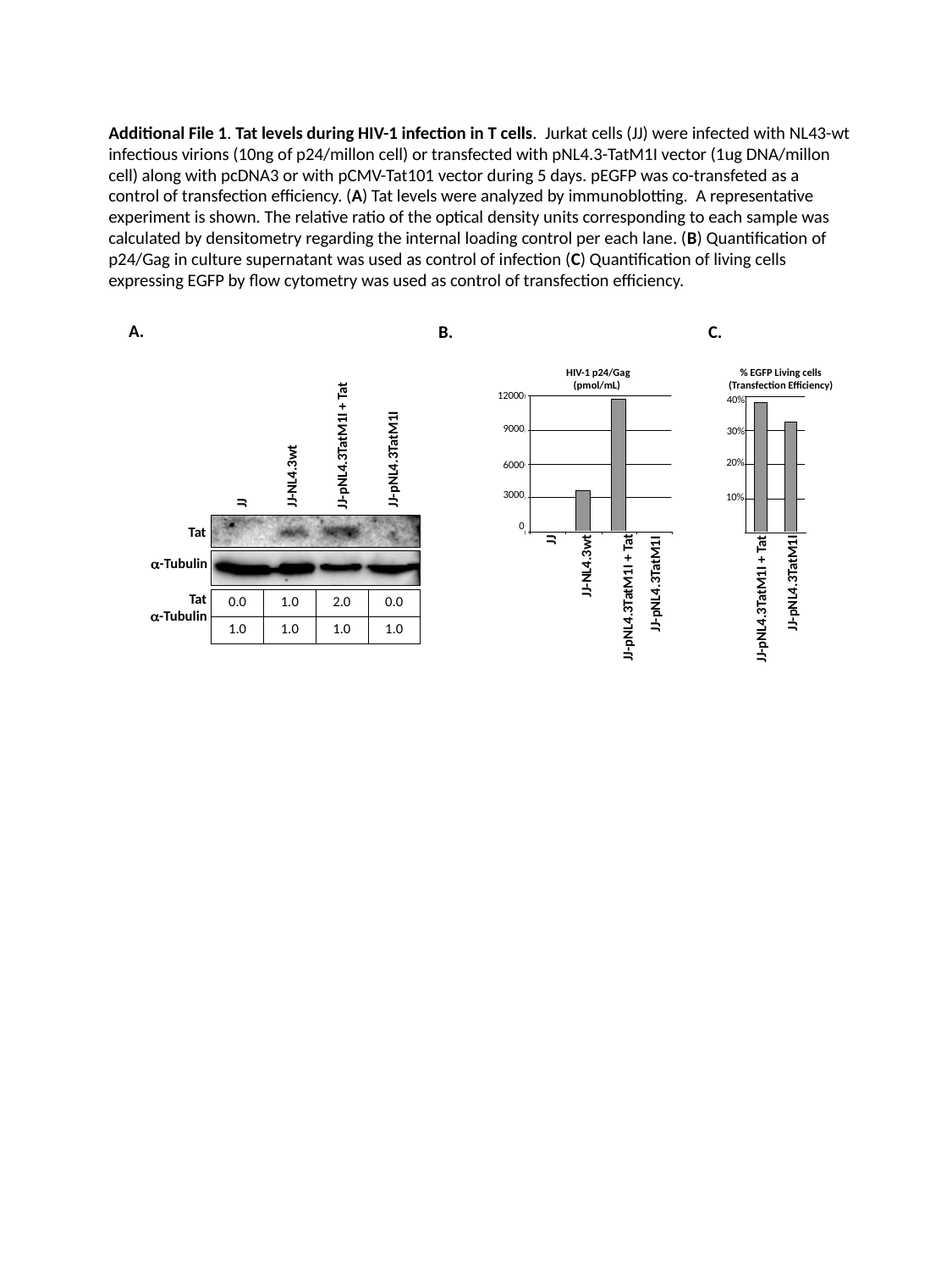

Additional File 1. Tat levels during HIV-1 infection in T cells. Jurkat cells (JJ) were infected with NL43-wt infectious virions (10ng of p24/millon cell) or transfected with pNL4.3-TatM1I vector (1ug DNA/millon cell) along with pcDNA3 or with pCMV-Tat101 vector during 5 days. pEGFP was co-transfeted as a control of transfection efficiency. (A) Tat levels were analyzed by immunoblotting. A representative experiment is shown. The relative ratio of the optical density units corresponding to each sample was calculated by densitometry regarding the internal loading control per each lane. (B) Quantification of p24/Gag in culture supernatant was used as control of infection (C) Quantification of living cells expressing EGFP by flow cytometry was used as control of transfection efficiency.
A.
B.
C.
JJ-pNL4.3TatM1I + Tat
JJ-pNL4.3TatM1I
JJ-NL4.3wt
JJ
% EGFP Living cells (Transfection Efficiency)
HIV-1 p24/Gag (pmol/mL)
12000
40%
9000
30%
20%
6000
3000
10%
0
Tat
JJ
α-Tubulin
JJ-pNL4.3TatM1I + Tat
JJ-pNL4.3TatM1I + Tat
Tat
JJ-NL4.3wt
JJ-pNL4.3TatM1I
JJ-pNL4.3TatM1I
| 0.0 | 1.0 | 2.0 | 0.0 |
| --- | --- | --- | --- |
| 1.0 | 1.0 | 1.0 | 1.0 |
α-Tubulin
