# Supplementary material for: Intracellular expression of Tat alters mitochondrial functions in T cells: a potential mechanism to understand mitochondrial damage during HIV-1 replication
Source: Retrovirology. 2015 Sep 16;12:78. doi: 10.1186/s12977-015-0203-3 (PMC4571071; doi:10.1186/s12977-015-0203-3)
Supplement: Supplementary file 2 — Additional file 2: Table S1. List of oligonucleotides designed for mtDNA amplification. [file 12977_2015_203_MOESM2_ESM.docx]

**Table S1**. Oligonucleotide list.

| **Oligonucleotide Name** | **Oligonucleotide Sequence** |
| --- | --- |
| MFN2‑s | 5’‑AGCAGTGGGAAGAGCTCTGTTA‑3’ |
| MFN2‑as | 5’‑TGCCCTTCACATGGACAAAGAT‑3’ |
| DNM1L‑s | 5’‑TGTTTTCAGAGTCATGGAGGCGCT‑3’ |
| DNM1L‑as | 5’‑ACAACAGGAGAAGAAAATGGGGTGGA‑3’ |
| COXII‑s | 5’‑TACAAGACGCTACTTCCCCTATCATA‑3’ |
| COXII‑as | 5’‑AACATCTCAGACGCTCAGGAAATA‑3’ |
| MTND2‑s | 5’‑TAAATAAGCTATCGGGCCCATA‑3’ |
| MTND2‑as | 5‑’ACCATCTTTGCAGGCACACTCA‑3’ |
| MTND5-s | 5‑’TCCACTCAAGCACTATAGTTGT‑3’ |
| MTND5-as | 5‑’TATTCCTGCTAATGCTAGGCT‑3’ |
| MTND6-s | 5‑’ TTCTGTTGAGTGTGGGTTTAGT‑3’ |
| MTND6-as | 5‑’ TCACCAAGACCTCAACCCCT‑3’ |
| MTCYB-s | 5‑’ TTAACCACTCATTCATCGACCT ‑3’ |
| MTCYB-as | 5‑’ TATAGGCCTCGCCCGATGTGT ‑3’ |
| MTCO3-s | 5‑’ TCCATAACGCTCCTCATACTAGGCCT‑3’ |
| MTCO3-as | 5‑’TACGGATGTGTTTAGGAGTGGGACTT ‑3’ |
